# COVID-19 Pandemic and Physical Exercise: Lessons Learnt for Confined Communities

**DOI:** 10.3389/fpsyg.2021.618585

**Published:** 2021-05-05

**Authors:** Amine Ghram, Nicola Luigi Bragazzi, Walid Briki, Yaser Jenab, Mehdi Khaled, Monoem Haddad, Karim Chamari

**Affiliations:** ^1^Department of Exercise Physiology, Faculty of Physical Education and Sport Sciences, University of Tehran, Tehran, Iran; ^2^Department of Cardiac Rehabilitation, Tehran Heart Center, Tehran University of Medical Sciences, Tehran, Iran; ^3^Laboratory for Industrial and Applied Mathematics, Department of Mathematics and Statistics, York University, Toronto, ON, Canada; ^4^Department of Physical Education, College of Education, Qatar University, Doha, Qatar; ^5^Department of Interventional Cardiology, Tehran Heart Center, Tehran University of Medical Sciences, Tehran, Iran; ^6^Independent Physician (Internal Medicine), Singapore, Singapore; ^7^Aspetar, Qatar Orthopaedic and Sports Medicine Hospital, Doha, Qatar

**Keywords:** coronavirus, physical activity, exercise, incarcerated, prison, health, sedentary

## Abstract

The novel pandemic called “Coronavirus Disease 2019” (COVID-19), as a global public health emergency and global threat, has affected many countries in unpredictable ways and impacted on physical activity (PA) behaviors to various extents. Specific populations including refugees, asylum seekers, and prisoners, are vulnerable groups with multiple complex health needs and worse health outcomes with respect to the general population worldwide and at high risk of death from the “Severe Acute Respiratory Syndrome-related Coronavirus type 2” (SARS-CoV-2). Governments around the world have been implementing preventive healthcare policies, including physical and social distancing, isolation, and confinement, to mitigate against the burden imposed by the COVID-19 outbreak. This pandemic period is characterized by reduced or lack of movement. During this period of lockdown, PA can represent an immunotherapy and a preventative approach to avoid the harmful effects of inactivity due to the pandemic. Moreover, PA could be prescribed to improve the immune system of specific populations (refugees, asylum seekers, and prisoners), which particularly experience the condition of being confined. The present narrative review discusses the potential impacts of COVID-19 pandemic on these specific populations’ health status and the importance of performing PA/exercise to reduce the deleterious effects of COVID-19 pandemic. In addition, we aim to provide useful recommendations on PA/exercise for these specific populations to maintain their level of independence, physical, and mental health as well as their wellbeing.

## Introduction

Viral infections can have a profound effect on human life, in terms of morbidity and fatality rate. One of the most lethal outbreaks in the history of mankind is the 1918–1919 Spanish influenza pandemic (Kain and Fowler, 2019). Even though influenza is a commonly widespread respiratory infection, various serious complications, such as pneumonia, can occur if the disease is not quickly and effectively controlled (Kohut et al., 2004). Worldwide, according to some estimations, seasonal influenza can cause up to 3–5 million cases of severe illness, and result in approximately 500,000 deaths per year due to the increased susceptibility among populations, especially those vulnerable (Cao et al., 2011). Recently, in late December 2019, a pneumonia outbreak of unknown etiology started in the city of Wuhan (Hubei province, mainland China) and has spread rapidly to neighboring provinces in China and around the world. Human-to-human transmission of the virus, initially denied, has been subsequently acknowledged (Chan et al., 2020). As of February 14, 2021 (15:29 GMT), the total number of confirmed cases has significantly increased up to 109,189,351 in the world, with 2,407,133 deceased and 81,230,179 recovered. This infection has been named as “Coronavirus Disease 2019” (COVID-19), whereas the infectious agent has been termed as the “Severe Acute Respiratory Syndrome-related Coronavirus type 2” (SARS-CoV-2).

This novel pandemic, as a global public health emergency and a global threat, has affected many countries in unpredictable ways and impacted physical activity (PA) behaviors to various extents. Particularly, people’s lifestyles and behaviors, such PA and sedentary behavior (SB) have been drastically affected due to the prolonged closures, isolation/confinement and physical and social distancing practiced during the COVID-19 pandemic. The still ongoing outbreak poses, indeed, challenges for all the population and, in particular, for specific populations including asylum seekers, refugees, and prisoners as the pandemic sweeps across the globe. The spread of the COVID-19 pandemic is threatening people’s health and life safety and has critical impact on social life and national economy, at the same time (Wan et al., 2020). The aftermath of such outbreaks does not only harm physical but psychological and social health as well (Li S. et al., 2020). The issue has become increasingly relevant as an increasing number of incarcerated individuals test positive for COVID-19 (Stephenson, 2020). People residing in areas characterized by high population density or rural areas with poor access to health care facilities, living in shared or congregate housing and multi-generational households, immigrants, refugees, racial and ethnic minorities are at higher risk for contracting COVID-19 (Ainsworth and Li, 2020). Moreover, multiple wave outbreaks are occurring due to the relaxing/easing of the public health measures implemented and the insurgence of new coronavirus strains racing across many countries (Siu et al., 2020). Being confined, isolated, hospitalized, or incarcerated is likely to compromise human wellbeing. SB (e.g., prolonged sitting or screen time), as a global public health concern, represents a major risk to wellbeing (Katzmarzyk et al., 2009; Dunstan et al., 2010; Ghram et al., 2020a) and is related to the adoption of unhealthy behaviors and lifestyles (e.g., physical inactivity, and dietary habits) (Bao et al., 2020). Thus, decreasing SB, adopting good personal hygiene practices, and regular PA and exercise may be an effective way to counteract/mitigate the detrimental effects of living under restricted environments due to COVID-19 spread. The common saying, “prevention is better than cure,” still holds true (Ghram et al., 2020a). Due to the lack of an appropriate antiviral agent and due to the scarcity of vaccine stocks, we need a safe intervention that can be globally implemented (Ramalingam et al., 2020). The European Union (EU), the United States (USA) and Canada, and Australia have significantly invested in sports-related programs and interventions aimed at engaging refugees and asylum seekers in sports-related and physical activities for health, therapeutic, or societal purposes (Spaaij et al., 2019). Indeed, PA and exercise could alleviate and mitigate against the symptoms and enhance the physical and psychological health status of individuals who are confined or incarcerated and exercise prescriptions could provide advice for these specific populations to be involved in suitable physical exercises and activities in order to promote the physical wellness as well as work efficiency (Ghram et al., 2020a). If, on the one hand, PA and exercise may facilitate the virus spread and, as such, should always be practiced under certain circumstances and strictly following the protective measures against COVID-19, on the other hand, PA and exercise might strengthen the immune system that could help to combat COVID-19 in these vulnerable populations (Ghram et al., 2020a; Laddu et al., 2020).

In the present narrative review, we aim to stress the importance of the public health measures that have been enforced to limit the COVID-19 spread and their effects among asylum seekers, refugees, and prisoners. Then, we focus on the risks of being isolated, physical inactivity and SB associated with the novel coronavirus outbreak. Finally, we discuss the consequences of being active, and the potential role of PA/exercise in counteracting the negative effects of the COVID-19 induced lockdown and reducing the risk of developing health problems among specific populations.

## What Is COVID-19?

Coronaviruses are enveloped, single-stranded, positive-sense RNA viruses that generally cause mild respiratory symptoms and occasionally severe and lethal infections (Fehr and Perlman, 2015). In the last decades, several large-scale coronaviruses-induced outbreaks have occurred, including the “Severe Acute Respiratory Syndrome” (SARS) outbreak in China (2003), and the “Middle Eastern Respiratory Syndrome” (MERS) outbreaks in the Kingdom of Saudi Arabia (2012) and in South Korea (2015) (Bok et al., 2020).

The current pandemic caused by SARS-CoV-2 can lead, as previously mentioned, to an usually mild but sometimes life-threatening infection (Wang et al., 2020), like bilateral pneumonia, myocardial injury, respiratory failure, acute respiratory distress syndrome (ARDS), multi-organ failure (MOF) and, in some cases, can even result in death (Holshue et al., 2020). Symptoms at the onset of COVID-19 infection as reported in hospitalized patients in China include fever, cough, fatigue, myalgia, sputum production, dyspnea, oppression in the chest, diarrhea, headache, anorexia, chest pain, sore throat, dizziness, palpitations, and vomiting (Gilani et al., 2020).

## Mode and Transmission of the COVID-19 Virus

Coronavirus Disease 2019 virus is mainly transmitted through respiratory droplets and physical contact routes (Burke et al., 2020; Chan et al., 2020; Huang et al., 2020; Li Q. et al., 2020; Liu et al., 2020). Droplet transmission can occur when a subject is in close contact with an individual with respiratory symptoms (e.g., coughing or sneezing) and is at risk of having his/her mucosae (mouth and nose) or conjunctiva (eyes) exposed to potentially infective respiratory droplets. Viral spreading may also occur via fomites surrounding the infected person (Ong et al., 2020).

During the early stages of the outbreak, Africa has shown very few COVID-19 cases compared to the number of cases reported in European and Asian countries, which can be explained by differences in the number of swabs performed, in the large-scale testing and reporting, as well as by the low volume of travel connections, enhanced border screening policies and the beneficial impact of Africa’s hot climate (Otu et al., 2020).

## Preventive Healthcare Measures of COVID-19 in the Community

Due to the highly contagious nature of the virus, it has rapidly spread globally, and many countries are experiencing overwhelmed and strained national health systems. Moreover, they are suffering from shortage of protection devices and human resources needed to adequately treat infected patients (ECDC, 2020). To cope with virus outbreak, the health care authorities have made dreadful decisions. Given the absence of effective therapeutic interventions at the time of the virus global spread, cities and countries have decided to adopt non-pharmacological measures, implementing self-isolation, social distancing, quarantine and even lock-down of entire communities and territories as well as travel restrictions (Wilder-Smith and Freedman, 2020).

According to the “World Health Organization” (WHO), several low-cost non-medical countermeasures that would contain spread have been recommended widely, including personal protective equipment like masks and gloves, reduction in the need for attending crowded public spaces, enhanced hygienic measures like frequent cleaning of door handles and other public surfaces, and similar measures (Bell et al., 2006).

While these measure, on the one hand, have contributed to saving lives, on the other hand, the COVID-19 pandemic is resulting in serious psychological effects such as anxiety, insomnia, panic behavior, fear, and hopelessness (Ammar et al., 2020b, 2021; Chtourou et al., 2020; Khan et al., 2020) and has caused and is still causing a massive economic shock across the world due to business interruptions, disruption and closures due to social-distancing measures (Martin et al., 2020). It was shown that COVID-19 home confinement has a negative effect on social participation and life satisfaction (Ammar et al., 2020a). To prevent viral infection, infected people should isolate themselves, which may increase the stress burden on family members due to the fear of getting infected (Khan et al., 2020).

In other words, preventive measurements during the massive lockdown may increase stress in the general and specific populations. Isolating infected individuals inside homes or jails/prisons may also increase the stress burden on family members due to the fear of getting infected. Indeed, the population must be informed during and after control of the pandemic about the possible impact of the viral outbreak, in terms of individual risk, outcomes of negative health behaviors, to take specific measures to mitigate such detrimental effects. Summarizing, public awareness regarding necessary actions to prevent the spread of a viral infection and counteract its negative impact is of paramount importance (Khan et al., 2020).

## COVID-19 Outbreak’s Impact on Refugees and Asylum Seekers

Asylum seekers and refugees experience severe mental and physical strain (war, violence, political and religious persecution, poverty, imprisonment, torture as well as traumatic and hard living conditions, economic hardship, discrimination, social exclusion, exploitation) (Knappe et al., 2019). People in refugee camps live in a closed environment and in close proximity that facilitate transmission of diseases (WHO, 2020). They also have a greater underlying burden of disease and worse health conditions than the general population, and frequently face greater exposure to risks such as smoking, poor hygiene and weak immune defense due to stress, poor nutrition, or existing diseases (WHO Regional Office for Europe, 2020). People in places of detention should have comprehensive awareness of COVID-19 prevention strategies, including adherence to hand hygiene measures, respiratory etiquette (covering coughs and sneezes), practicing physical distancing (at least 1 meter from others), being alert to signs and symptoms of COVID-19, staying away from ill people (in the case of staff), and staying home when ill.

Many asylum seekers have complex mental health needs which can be exacerbated by the challenging circumstances in which they live and difficulties accessing health services (Haith-Cooper et al., 2018). Refugees have a higher prevalence rate for psychopathological disorders such as post-traumatic stress disorder (PTSD), depression, or anxiety disorders (Gerritsen et al., 2006). All this together makes COVID-19 spreading in world’s refugee camps particularly concerning. Millions of displaced people in Bangladesh (Rohingya refugees), Syria (Idlib province), Lebanon, Africa (Kenya, Libya), and France (northern France), amongst others, have been gathered into crowded refugee camps and makeshift settlements, and threatened by the coronavirus spread (UNHCR, 2020).

In Bangladesh, refugee volunteers and aid workers have been struggling to educate people in these camps about the coronavirus to limit the spread of the virus (UNHCR, 2020). In Syria, families have been moved from large communal tents to individual tents, but social distancing is almost impossible (UNHCR, 2020). In Kenya, the United Nations High Commissioner for Refugees (UNHCR) has reduced contacts between residents and humanitarian workers (UNHCR, 2020). In addition to closing schools, some businesses and markets, and imposing curfews, UNHCR has been urging governments to include refugees and asylum-seekers in national plans to combat COVID-19 in Libya (UNHCR, 2020). In France, French authorities transferred up to 2,100 refugees and migrants in Calais and Dunkirk from informal camps to accommodation centers where they would be expected to follow the same coronavirus lockdown rules as the rest of France (UNHCR, 2020). In Lebanon, many steps have been taken to distribute soap, set-up isolation units and support Lebanon’s health system to set up more intensive care units (UNHCR, 2020).

Refugees, asylum seekers, and displaced persons having fled the horrors of war, terrorism, and persecution, want to re-build their lives, and become a part of their new community. They have to face difficult living conditions, economic hardship, discrimination, social exclusion, and exploitation and, as mentioned before, are often exposed to severe mental and physical strain due to war, violence, political and religious persecution, poverty, imprisonment, or torture (Xin et al., 2017). Forced migration and resettlement are associated with distressing circumstances that contribute to poor physical and psychological wellbeing including high rates of trauma and other stress-related disorders (Ley et al., 2018; Ley and Barrio, 2019). Also, physical inactivity is a common phenomenon among refugees and immigrant populations (Crespo, 2000; Gerber et al., 2012). Refugee/asylum seeker status may affect the decision to engage with PA and/or exercise due to competing priorities, the temporary nature of living and mental and physical health (Haith-Cooper et al., 2018).

## COVID-19 Outbreak’s Impact on Prisoners

The COVID-19 outbreak can occur easily in environments such as the jail and prison that represent shared or congregate living environments, due to the higher number of people working or residing in correctional facilities. Jails and prisons are constructed to maximize public safety to prevent the transmission of disease or to efficiently deliver health care. However, jails and prisons often lack sufficient hand washing areas, isolation rooms, and personal protective equipment (Bick, 2007) that may increase the probability of transmission of the virus amid inmates. Visits to prisons are temporarily suspended in many countries worldwide (Hewson et al., 2020) and the loss of such visits could lessen the use of social support for mitigating against and coping with mental distress and the risk of suicide and self-harm among prisoners (De Claire and Dixon, 2017).

As the virus continues to spread, the public health response to the pandemic in correctional facilities becomes more and more challenging and warrants a holistic approach. Attempts to control the COVID-19 community transmission are not likely to succeed if strict infection prevention and control measures, including testing, treatment, and care, are not implemented in prisons and other places of detention as well. As part of the response, the WHO has worked with relevant institutions and stakeholders to develop guidelines on preparedness, prevention, and management of COVID-19 in prisons as well as in other places of detention.

In addition, correctional facilities should provide sanitary guidelines to inmates who are exposed to air-borne droplets during staying in their cells or family visiting. Inmates, who tested positives and have the risk to transmit the virus through culinary activities in prison, should obviously be isolated and medically controlled and be careful regarding how to store, prepare and eat perishable food in their cells (CDC, 2020).

Staying home is not an option for people who are in prisons and jails across the world and social distancing is nearly impossible to practice except in solitary confinement. This results in dramatically impacting mental and physical health (Reiter et al., 2020). According to Human rights agencies, long-term solitary confinement is a torture (Reiter et al., 2020) and refers to the physical and social isolation of an individual which can induce psychological harms of segregation including self-harm, anxiety, depression, paranoia, and aggression (Kaba et al., 2014; Haney, 2018). Isolated prisoners would typically spend their time in a single cell for 22.5–24 h a day and solitary confinement can last for months or years, and can be of an indeterminate duration which can promote a sense of helplessness and increase hostility and aggression (Shalev, 2008).

Incarcerated inmates in jail facilities have an increased risk of human immunodeficiency virus infection, hepatitis B virus infection, hepatitis C virus infection, syphilis, gonorrhea, chlamydia, and *Mycobacterium tuberculosis* infection (Hammett et al., 2002), the acquisition of infection with airborne organisms, such as *M. tuberculosis*, influenza virus, and varicella-zoster virus (Bick, 2007), as well as high rates of hypertension (Binswanger et al., 2009; Trotter et al., 2018), poor sleep quality (Harner and Budescu, 2014), anxiety, and depression (Binswanger et al., 2010).

Practicing social distance in prisons is difficult due to the issues of overcrowding and the fear increases if correctional facilities become epicenters in the coronavirus pandemic. Correctional facilities try to minimize the risk of transmission by quarantining a sick prisoner which is the segregation that is usually used as a punitive measure. Another way to protect inmates who are elderly, immunocompromised, medically vulnerable, close to release, or associated with minor, non-violent crimes, is to decarcerate or release them. Indeed, sending them home can protect prisoners, correctional workers, their families and the broader community. Upon release from prison, many individuals experience difficulty in maintaining good health (Wallace and Wang, 2020) because infected prisoners might bring disease from jail into their communities. Therefore, in case of pandemic, released prisoners should pass by a quarantine period before entering in contact with their family members and community.

Imprisoned people have usually a poor health status in comparison with the general population (Fazel and Baillargeon, 2011; Mannocci et al., 2015). More than 40 of the 50 largest clustered outbreaks in the United States have occurred in jails and prisons (Macmadu et al., 2020) and the number of COVID-19 cases is 5.5 times higher among people who are incarcerated (Saloner et al., 2020). Several behavioral factors including use of intravenous drug, alcohol misuse, smoking, and physical inactivity could increase the risk of morbidity, mortality, and mental disorders (Brinded et al., 2001; Cashin et al., 2008; Fischer et al., 2012; Battaglia et al., 2015). As well, overcrowding, and high occupant turnover may increase the risks of transmission in prisons and jails (Macmadu et al., 2020).

## PA/Exercise During COVID-19 Pandemic

As of today, clinical practice, community programs, mass-media campaigns, and population strategies have focused mainly on encouraging and supporting individuals to be more active, during staying at home. As the mortality rate of patients with positive COVID-19 test and confined and isolated people continues to increase and chronic medical problems from inactivity become increasingly prevalent, there is now an imperative to increase the PA levels of refugees, asylum seekers, and prisoners and overall total daily energy expenditure.

During the COVID-19 induced confinement, the behavior of prolonged sitting time that is a predictor of weight gain, requires that individuals should be aware of the amount of PA necessary to achieve better health outcomes, understand the importance of PA and exercise in relation to good physical and mental health, and prevent overweight, obesity and chronic disease (Owen et al., 2009). PA is defined as any bodily movement produced by skeletal muscles which results in energy expenditure and can be categorized into occupational, sports, conditioning, household, or other activities. Exercise is a planned and structured program of motor actions to improve or maintain components of physical fitness (Caspersen et al., 1985). As previously mentioned, PA/Exercise is a potent stimulus of immune function (Ghram et al., 2020a; Laddu et al., 2020) to fight against the mental and physical consequences of COVID-19 quarantine (Jiménez-Pavón et al., 2020). The types of exercise prescribed can vary by mode, dose, setting, the person who delivers the intervention, and any accompanying behavioral strategies (e.g. counseling, pamphlets) (Campbell et al., 2007). To attenuate negative effects of SB and physical inactivity, individuals are required to participate in regular PA and minimize the time they spend sitting to prevent cardiovascular and metabolic disease, certain types of cancer, and mental deficits (Vogel et al., 2009; Piercy et al., 2018).

Social and physical distancing and isolation are required to stop the transmission of infectious disease. Facing this new situation, exercise professionals encourage people to maintain good health and have recommended to use online technology to prescribe, and monitor exercise, such as mobile telephones messages, apps, email, video calls, or other internet-based strategies (de Oliveira Neto et al., 2020; Ghram et al., 2020a). Isolated/confined or imprisoned individuals should raise energy expenditure as much as is necessary by participating in moderate to vigorous PA to prevent obesity and decrease sedentary time. While the benefits of PA are not disputed, reducing sedentary time is of vital importance (Bowden Davies et al., 2019).

Physical exercise can be a major part of a multi-component strategy targeted at reducing psychological ill health in asylum seekers, refugees, and prisoners (Mannocci et al., 2017). Evidence shows that physical exercise is widely recommended for general and specific populations to maintain health status, improve daily life activities, play a fundamental role in protecting against disease, and provide biological and psychological benefits. The American College of Sports Medicine (ACSM) has released information on how to remain active during the COVID-19 pandemic (ACSM, 2020) and has emphasized the positive effects of the regular practice of physical exercise on the enhancement of the immunological system in humans, showing that physically active people have a lower risk of developing chronic-degenerative diseases, which is highly pertinent and related to COVID-19, as those affected are at higher risk if infected by SARS-CoV-2 (Pedersen and Saltin, 2015; de Oliveira Neto et al., 2020). Therefore, people in isolation and with a positive diagnosis for COVID-19, but who are asymptomatic, should be able to continue the regular practice of moderate intensity PA. However, in the presence of symptoms (e.g., fever, cough, and dyspnea), the practice of PA should be interrupted and medical assistance should be sought (Joy, 2020). Special attention should be paid to asylum seekers, refugees, and prisoners, because among these vulnerable populations PA and exercise have benefits on physical and mental health and can prevent psychological disorders.

## PA/Exercise as an Effective Countermeasure to the Psycho-Social Effects of COVID-19 Among Refugees and Asylum Seekers

Considering that asylum seekers and refugees represent highly heterogeneous groups with different cultural backgrounds and might be more prone to the psychosocial impact of the COVID-19 pandemic, PA/exercise might be an effective and safe alternative to mitigate this risk and reduce the breadth and depth of physical and mental needs of these vulnerable groups.

Among immigrants and those seeking refuge into highly developed nations, PA level is not satisfactory and, in general, lower than among non-immigrants (Sternfeld et al., 1999; Crespo et al., 2000; Gadd et al., 2005), and programs aimed at increasing PA within 10 years of arrival may be particularly effective (Goel et al., 2004). Exercises and sports-related activities can be utilized to properly treat various psychiatric diseases (Lam and Riba, 2016), and are instrumental to human wellbeing (Lederman et al., 2017). Even though international organisms, such as the UNHCR, acknowledge the feasibility of exploiting exercise and sport as a peace-building measure in refugee camps with healthy individuals (Korsik et al., 2013), evidence has shown that adequate PA levels have also a beneficial impact on traumatized subjects (Rosenbaum et al., 2015, 2018).

Twelve weeks of biofeedback-based cognitive behavioral therapy (CBT-BF) combined with PA (including mixed activities such as flexibility, strength, and endurance training) resulted in enhanced coping strategies and mechanisms to face pain with respect to CBT-BF alone or a waiting-list control condition in 36 refugees from Germany and Switzerland reporting PTSD symptoms (Liedl et al., 2011). In another study from Denmark, Stade et al. (2015) reported high acceptability, compliance and satisfaction with basic body awareness therapy (BBAT). Positive effects on mental health outcomes were observed after 12 weeks of regular physical activity among Bosnian refugees in the United States (Xin et al., 2017). Regularly attending a 8-week sport and exercise program has been found to exert a positive influence on a wide range of mental health outcomes, including PTSD, depressive, anxiety symptoms, health-related quality of life, handgrip strength, and perceived and cardiorespiratory fitness (Knappe et al., 2019).

Moderate PA and/or sport can beneficially modulate physical and mental health and wellbeing for refugees and asylum seekers living in particularly challenging conditions. Refugees and asylum seekers should be instructed about accumulating adequate PA levels through changes in their lifestyle [for example, by walking faster or increasing intensity when carrying out housework (Haith-Cooper et al., 2018)]. Thus, performing PA during the COVID-19 pandemic could potentially help these populations better cope with their limited movement and difficult psychological conditions.

## PA/Exercise as an Effective Countermeasure to the Psycho-Social Effects of COVID-19 Among Prisoners

Prisoners and inmates are at higher risk of adopting unhealthy lifestyles such as tobacco smoking, drug abuse, low physical activity and irregular dietary habits, factors that may lead to the insurgence of acute and chronic physical and psychological disorders (Mannocci et al., 2015).

Lack of PA may affect negatively psychological status of prisoners and inmates and increase anxiety (Boothby and Clements, 2000), stress, and depression (Plugge and Fitzpatrick, 2005) rates. One of the factors challenging their ability to practice PA and exercise is overcrowding (WHO Regional Office for Europe, 2020). Thus, prisoners experience difficulties in moving and undertaking the necessary amount of moderate PA necessary to benefit their health (WHO Regional Office for Europe, 2020). A “decent” prison regime should ensure that prisoners are able to be compliant with the WHO guidelines and recommendations on PA, and are sufficiently instructed how to make an informed choice about optimal PA and exercise programs and strategies (WHO Regional Office for Europe, 2020). It was shown that PA is a simple and cost-effective way to positively modulate the health-related quality of life (QoL) of prisoners and can contribute to achieving “healthy prison” objectives in practice (Meek and Lewis, 2012). Regular physical exercise can enhance inmates QoL, allow a better social cohesion and integration at the end of detention in prisons (Mannocci et al., 2015), reduce depression, anxiety, stress (Buckaloo et al., 2009), and sleep problems such as insomnia (Elger, 2009), increase muscle tone, strength, stamina (Nelson et al., 2006), and functional capacity (Battaglia et al., 2013), and improve overall physical fitness of incarcerated people (Pérez-Moreno et al., 2007).

Taking into consideration the health status of prisoners, the lack of availability of safe equipment for training, and the limited space derived from the condition of being incarcerated, we suggest to make a bodyweight workout plan to help the prisoner get ripped while being in isolation. Warm-up should last at least 10 min (jog on the spot, high knee tucks, butt kicks, skip, forward lunge and twist, carioca, lateral lunge and twist, slides, high knee crossovers, exaggerated backpedal). Other exercises can be performed easily such as Push-Up, Dips, Sit-Ups, Squats, Burpees, Star-Jumps, Wide-Grip Pull-Ups, and Step-Ups. Furthermore, relaxation techniques and stretching exercises are easy to carry out in a practical fashion without the risk of being exposed to a contaminated environment and are effective in promoting health gains and reduce stress levels (Bentlage et al., 2020; Ghram et al., 2020a). Stretching exercises may improve stability and balance of these vulnerable populations (Costa et al., 2009; Handrakis et al., 2010; Ryan et al., 2010; Chatzopoulos et al., 2014; Ghram et al., 2016, 2020b).

## Practical Recommendations

Coronavirus Disease 2019 outbreak poses significant challenges to these specific populations. Preventative healthcare measures should be implemented to reduce disease transmissions within prisons and camps and decrease negative mental health problems wherever possible. Since less attention for these specific populations compared to the general population is generally paid, we recommend that these vulnerable populations are potentially encouraged to stay more physically active, and informed about the risk of SB and physical inactivity. There is increasing need in this community to encourage prisoners, asylum seekers, and refugees to engage at least in low exercise intensity to enhance their fitness, physical and psychological health, and wellbeing and reduce the risk of disability. In further research, researchers and scientists are required to focus on developing more efficient strategies to reduce the negative effect of pandemic spread. Future studies should investigate the impact of PA and exercise in these vulnerable groups during pandemic outbreak, as well as after having achieved the full control of the spread of the epidemic in non-infected and infected individuals.

## Conclusion

The COVID-19 pandemic is still ongoing, exerting a dramatic, detrimental impact on PA-related patterns and sedentary lifestyle and posing severe challenges for asylum seekers, refugees, and prisoners, globally. The available body of evidence suggests that exercise is medicine and protects from infections, mainly among specific vulnerable and frail populations. Physical exercise, indeed, positively impacts mental wellbeing and provides protection to individuals against depression and anxiety as it causes biochemical and biophysical changes in the brain which can positively influence their mood. Since public health measures (confinement, isolation, social and physical distancing) are aimed to protect vulnerable populations during pandemic, but, on a long-term scale, may have secondary negative effects on health and well-being, these specific vulnerable populations should live in adequate physical conditions, having regular access to fresh, healthy air and being able to practice PA/exercise. Notwithstanding variations in individual tolerance to living in confined environments, there is remarkable evidence of the consistency of public health measures psychological as well as physiological health effects. In addition, PA/exercise can be considered as a viable tool for the treatment, and management of people who are living in confined environments and/or are experiencing limited movement because of virus-related outbreaks.

## Author Contributions

AG, NLB, and KC conceived the manuscript. All authors wrote and revised the manuscript.

## Conflict of Interest

The authors declare that the research was conducted in the absence of any commercial or financial relationships that could be construed as a potential conflict of interest.
